# Tailoring Cardiac Synthetic Transcriptional Modulation Towards Precision Medicine

**DOI:** 10.3389/fcvm.2021.783072

**Published:** 2022-01-14

**Authors:** Eric Schoger, Sara Lelek, Daniela Panáková, Laura Cecilia Zelarayán

**Affiliations:** ^1^Institute of Pharmacology and Toxicology, University Medical Center Goettingen, Goettingen, Germany; ^2^DZHK (German Center for Cardiovascular Research), Partner Site Goettingen, Goettingen, Germany; ^3^Cluster of Excellence “Multiscale Bioimaging: From Molecular Machines to Networks of Excitable Cells”, University of Goettingen, Goettingen, Germany; ^4^Max Delbrück Center for Molecular Medicine in the Helmholtz Association, Berlin, Germany; ^5^DZHK (German Center for Cardiovascular Research), Partner Site Berlin, Berlin, Germany

**Keywords:** single cell sequencing, CRIPSR/Cas9 system, endogenous gene activation, cardiomyocytes, gene regulation

## Abstract

Molecular and genetic differences between individual cells within tissues underlie cellular heterogeneities defining organ physiology and function in homeostasis as well as in disease states. Transcriptional control of endogenous gene expression has been intensively studied for decades. Thanks to a fast-developing field of single cell genomics, we are facing an unprecedented leap in information available pertaining organ biology offering a comprehensive overview. The single-cell technologies that arose aided in resolving the precise cellular composition of many organ systems in the past years. Importantly, when applied to diseased tissues, the novel approaches have been immensely improving our understanding of the underlying pathophysiology of common human diseases. With this information, precise prediction of regulatory elements controlling gene expression upon perturbations in a given cell type or a specific context will be realistic. Simultaneously, the technological advances in CRISPR-mediated regulation of gene transcription as well as their application in the context of epigenome modulation, have opened up novel avenues for targeted therapy and personalized medicine. Here, we discuss the fast-paced advancements during the recent years and the applications thereof in the context of cardiac biology and common cardiac disease. The combination of single cell technologies and the deep knowledge of fundamental biology of the diseased heart together with the CRISPR-mediated modulation of gene regulatory networks will be instrumental in tailoring the right strategies for personalized and precision medicine in the near future. In this review, we provide a brief overview of how single cell transcriptomics has advanced our knowledge and paved the way for emerging CRISPR/Cas9-technologies in clinical applications in cardiac biomedicine.

## Introduction

Cardiovascular disease, a leading global cause of death, is accounting for 17.3 million deaths per year; a number that is expected to grow to more than 23.6 million per year by 2030 (1). Common cardiovascular disease conditions leading to heart failure include myocardial ischemia or cardiomyopathies such as ventricular hypertrophy that can be inherited or stress-induced. These conditions result among others in deficient oxygen supply, eventually affecting cardiomyocytes' transcriptional and differentiation states that precede substantial cardiomyocyte loss due to the lack of regenerative capacity. Simultaneously, non-cardiomyocytes also undergo significant phenotypic and transcriptional changes leading to imbalanced intercellular communication. Comorbidities, including hypertension, dyslipidemia, and glucose imbalance, produce a hostile environment that modifies disease progression increasing heart failure risk (2). Collectively, these factors determine the extent of myocardial damage, which has a direct impact on patient specific disease progression while also depending on time of injury (3). Hence, a heterogeneous tissue remodeling characterizes heart failure progression. Current therapeutic guidelines for cardiovascular diseases involve reperfusion-based treatments and drug therapies mostly alleviating the symptoms generated by cardiac functional deterioration (1, 4–6). Therapeutic concepts targeting favorable reparative myocardial remodeling remain challenging. A big problem lies in the inefficient regenerative capacity of the heart. Based on the studies of various animal models as well as human data on cardiac regeneration, the attention has recently shifted toward the possibility of repairing diseased hearts by reawakening the intrinsic regenerative potential (7). The analysis of the integration of 14C generated by nuclear bomb tests during the Cold War allowed to estimate that fewer than 50% of cardiomyocytes are physiologically exchanged during the course of life in the human heart (8), indicating the intrinsic potential of cardiomyocytes renewal in the human myocardium. Indeed, in response to heart injury, the rate of cardiomyocyte cell cycling increases in the peri-infarct region; however, this is far too limited to effectively replace the lost cardiomyocytes (9). Thus, efforts have been made toward stimulating cardiomyocytes proliferation based on factors responsible for the transient neonatal heart regeneration in animal models. To promote endogenous cardiomyocyte proliferation, initial approaches targeted universal cell cycle regulators such as cyclins, cyclin-dependent kinases (CDKs), tumor suppressor genes, and cell-intrinsic signaling pathways that regulate cardiomyocytes proliferation during development (7, 10). These include mainly developmental transcription factors comprising the Hippo, Hedgehog (HH), Wnt pathway, HIF1α, SMADs, TBX20, p53, Jarid2, GATA4, MEIS1/2, Retinoblastoma, PITX2, E2F family members, KLF1, REST (11–25) as well as chromatin remodeling proteins (26), and microRNAs (miR-590, miR-199a, miR-548c, miR-509, miR-23b, miR-17-92 cluster, miR302-367, miR-143) (27–30). Recently, induced expression of the pluripotency factors OCT4, SOX2, KLF4, AND C-MYC (OSKM) was shown to trigger cardiomyocyte dedifferentiation by reprogramming cardiomyocytes to a fetal-like regenerative state. Short-term OSKM expression ameliorated myocardial damage and improved cardiac function upon myocardial infarction (31). These findings serve as a proof-of-concept to unleash the intrinsic regenerative potential of cardiomyocytes.

Another challenge is the application of exogenous factors to the adult heart, while preventing aberrant cell proliferation (26). Secreted factors such as Neuregulin 1 (NRG1), an agonist for the ERBB2 and ERBB4 receptor tyrosine kinases and a key mitogen during heart development, were shown to promote the reactivation of cell cycle (32–34). NRG1 is reactivated in both, zebrafish and mouse heart regeneration, stimulating cardiomyocyte proliferation and metabolic reprogramming; a potentially less risky approach than direct overexpression of cell cycle modulators or kinases (33, 35–37). Although stimulating cardiomyocyte proliferation is a promising strategy to boost myocardial regeneration in adult hearts, several obstacles must be overcome before reaching clinical applications. These include, for instance, inefficient and uncontrolled cell proliferation with an increased risk of cancer (9). Furthermore, not only proliferation, but also cardiomyocytes maturation needs to be coordinately re-activated; which implies both structural remodeling and dramatic metabolic alterations driven by different mechanisms (38). In order to tackle this problem, the fundamentals of complex regulatory networks that govern cardiomyocyte regeneration and repair embedded in their pathophysiological environment, need to be understood in the disease context (39, 40). This will help to tailor therapeutic strategies correcting specific cellular defects.

A powerful tool capable of deciphering individual cellular responses within tissues is single cell sequencing (SCS). Using the SC transcriptomic data, the spatiotemporal interplay of different cell types within tissues enables to delineate dynamics during disease progression (41, 42). Further bioinformatic assessment of ligand–receptor interactions allows us not only to measure the expression of ligands and receptors in multiple cell types, but also to systematically decode intercellular communication networks that function in homeostasis and are altered in disease states (43). Having a better understanding of how exactly transcriptomic changes in both, healthy and pathological conditions mediate phenotypic effects at the SC resolution, will allow the use of synthetic transcription for correcting disease conditions. This can be achieved by using programmable nucleases such as DNA targeting class II clustered regularly interspaced short palindromic repeats (CRISPR)/Cas9 systems (44). These rapidly advancing technologies have expanded the applications of genetic research across the world by mediating transcriptional control of endogenous genes to model and, moreover, to treat common and multifactorial diseases in one-in-a-lifetime approaches in the near future. This review aims to summarize the current status toward deciphering the cardiac disease transcriptome and to describe the novel approaches in which molecular tools including CRISPR/Cas9 can be used to modulate and revert disease conditions.

## Real-Time Single Cell Transcriptome Profile of the Heart, the Basis of Precise Therapeutic Target Identification

The heart is composed of four morphologically as well as functionally distinct chambers, which requires an exact orchestration of all heterogeneous cell populations to guarantee its proper function (45). This is governed by a spatiotemporal pattern of gene regulation and cell-cell communication which, when altered upon disease condition, results in significant phenotypic changes and imbalanced intercellular communication. This leads to organ tissue remodeling triggering a vicious circle driving disease progression. Knowing crucial factors, which perturbation results in alteration of gene regulatory networks, maladapted cellular behaviors, and ultimately to a disease condition, will help to develop more efficient and tailored therapies. Despite decades of research, the medical interventions for treating cardiac abnormalities have not dramatically changed from the classical symptomatic treatment, remaining the clinical need unmet worldwide.

One of the critical advancements to understand disease states in the past years was the adaptation of several SCS platforms for profiling all heart cells' transcriptome. Even though application of SCS for many tissues was rapidly and successfully adapted, the use of this method in the heart remained quite challenging for some time. While isolation and sequencing of several non-cardiomyocyte cell populations, including immune and endothelial cells, were relatively straightforward, singularization and dispensing of large, elongated cardiomyocytes for sequencing were challenging. To circumvent this issue, single nuclei sequencing (SNS) has been adapted for cardiomyocytes as well as recent advances have been made toward whole cell sequencing (46–48). These technological innovations have allowed accurate use of SCS for all heart cells embedded in a tissue including cardiomyocytes. The different platforms used for SCS of the mammalian heart including human are well-described elsewhere (46, 47), and are not the focus of this review; only few examples of their application for heart tissue and their potential therapeutical implications will be discussed. Several studies provided comprehensive transcriptomic data that can be used to extract valuable information of diseased vs. healthy heart. Litvinuková et al. (41) provided comprehensive transcriptomic data on six distinct cardiac regions of the healthy adult heart deploying SNS of cardiomyocytes and SCS of enriched fibroblast, stromal, vascular and immune cell populations. An important observation in this study was the identification of cardiomyocyte population heterogeneity among the atrial and ventricular compartments (41). This study highlighted not only chamber-specific and lineage-specific profiles, but also sex differences of the healthy heart.

Other heart-disease oriented studies have focused on the cellular composition upon ischemic injury in the murine heart (42, 49, 50). SCS of the interstitial cell population showed comprehensive dynamics of cardiac stromal, vascular and immune cells of healthy and ischemic hearts. A novel activated fibroblast population characterized by an anti-WNT signaling transcriptome signature was identified (49). This particular observation is advancing our understanding of the role of transcriptional activation of WNT effectors and inhibitors, previously observed in the whole heart tissue upon stress (51). WNT signaling plays a complex role in cardiac biology and disease, affecting different cell types including cardiomyocytes, fibroblasts, and endothelial cells (51). Many drugs inhibiting WNT signaling are currently under investigation for their potential impact in heart repair (49, 51, 52). It is therefore pivotal to identify the cell-specific transcriptional profiles in order to target the correct cell population. A transient phenotypic change has been identified upon ischemic injury in a murine model. In this model, endothelial cells undergo a transient mesenchymal activation within the first days after myocardial damage, but do not acquire a long-term mesenchymal fate (42). The authors concluded that the transient mesenchymal fate of endothelial cells may facilitate cell migration and clonal expansion to promote regeneration of vascular networks (42). These data indicate the intrinsic regenerative potential of the heart, which is rendered inefficient in long-term remodeling and can be used as regenerative therapeutic targets. Using a different protocol, SCS was also performed using infarct and border zone regions and was compared to control hearts (50). Similar to other studies, they could detect cell type-specific upregulation of various genes between healthy and diseased subpopulations of various cell types. In this study, *Ckap4* was reported as a novel marker specifically upregulated in activated fibroblasts. The authors further identified a subset of epicardially located cardiomyocytes expressing *Myoz2*, a protein that tethers α-actinin to the hypertrophy inducer calcineurin, thereby inhibiting hypertrophic response (53). This indicates that subpopulations of cardiomyocytes respond differently to known hypertrophic factors, and would limit therapies that assume homogenous hypertrophic response among cardiomyocytes populations.

Yekelchyk et al. specifically investigated the transcriptional profile of mono and multi-nucleated adult cardiomyocytes under baseline conditions and in pressure-induced cardiac hypertrophy in the murine heart (48). Using an image-based quality control system and strict exclusion criteria, they concentrated on rod-shaped adult cardiomyocytes. This differs from other studies using the same system (3, 41). A noteworthy observation of this study is the elucidation of cardiomyocytes clusters correlating with the expression of basic helix-loop-helix transcription factor HIF1α, a master regulator of hypoxic stress response which was the main driver of heterogeneity in this pathological condition. This is in line with CreERT2-based lineage-tracing studies revealing a population of hypoxic cycling cardiomyocytes resembling neonatal proliferative cardiomyocytes that contribute to the slow cardiomyocyte turnover occurring in the adult mammalian heart (54). Interestingly, overexpression of a downstream target of HIF1α, the Zinc finger E-box-binding homeobox 2 (ZEB2), improves cardiomyocyte survival and cardiac function as well as angiogenesis following cardiac damage (55). Activation of HIF1α expression is well described in pathological conditions (56) now the prospective analyses of its activation in distinct cell types will offer a new perspective to interfere with pathological phenotypes in a cell-dependent manner, which may be applicable to other transcription factors. Indeed, HIF1α regulates cardiac fibroblasts activation upon ischemic injury by limiting their proliferative capacity (57), highlighting even more the necessity for cell-targeted therapies.

Furthermore, Wang et al. (3) have analyzed heart cells and their interconnection on normal healthy and patients with heart failure as well as those with functional recovery after treatment with a left ventricular assist device (LVAD) at single-cell resolution. Applying bioinformatic tools, the authors studied transcription-factor-centered regulatory networks and evaluated regulon activities in cardiomyocytes. They demonstrated regulation of transcription factor-depending regulons such as JUN, CEBPD and TCF7L2, which were previously described in disease conditions (58–60). However, the data exhibited distinct profiles of regulation among the different conditions, strongly indicating the presence of specific target disease-induced pathways and networks, warranting more disease-specific treatment of cardiovascular diseases. Wang et al. also provided evidence supporting a model, in which non-cardiomyocytes undergo substantial changes during the loss of normal heart function that may direct disease progression and prognosis. The comparison of cell types of diseased and healthy hearts led them to identify non-cardiomyocyte cell types necessary for maintaining myocardial homeostasis and for protecting the heart tissue from failing. As an example, based on the data they obtained, they performed transplantation of ACKR1+ endothelial cells into the ischemic heart, which significantly enhanced cardiac function (3). More importantly, they showed that transcriptome profiles of all cell populations from the patients with improved heart function shifted considerably toward normal physiological state. Thus, this observation implies the plasticity and substantial recovery potential of cardiac cells in the adult human heart, even in end-stage heart failure (3). This data holds promise for the development of strategies that can reverse disease cell-states by exploiting endogenous recovery programs of the heart. Using SCS analysis in cardiac biopsy samples from patients with heart failure before treatment, the presence of failing cardiomyocytes characterized by the activation of DNA damage response genes only in patients showing poor prognosis was validated (61, 62). Hence, these methods present a realistic approach to determine clinical prognosis and treatment response.

Another study analyzed human left ventricular samples including control non-failing, hypertrophic and end-stage cardiomyopathy as well as heart failure samples along with mouse hearts at different stages after experimentally-induced pressure overload to investigate the pathological progression of cardiac hypertrophy (63). Specifically, their findings suggested a pivotal role of macrophage subtype switching toward an inflammatory state upon reduction of cardiac function during pathological cardiac hypertrophy. Therefore, they tested the effectiveness of the anti-inflammatory treatment on the stage-specific macrophages between 2 and 5 weeks after induced pressure overload. This resulted in ameliorated cardiac hypertrophy. However, an earlier anti-inflammatory treatment before 2 weeks failed to avoid decline in cardiac function (63). This suggests that stage-specific targeting of macrophages may serve to suppress pathological cardiac hypertrophy and influence the course of disease progression. This study also revealed conserved cellular and molecular basis of cardiac hypertrophy between mouse and human, providing an excellent platform to investigate mechanisms that can be translated toward improved therapies. The extraction of (sub-)cell types and their respective transcriptome profiles allows for analyses of differential gene and gene cluster expression as well as gene regulatory networks in health and disease states. This will help to exactly specify their role for targeted functional phenotyping (64). For instance, ischemic and non-ischemic human heart samples subjected to SNS yielded a catalog of cardiomyocyte and non-cardiomyocyte (vascular endothelial cells, endocardial endothelial cells, fibroblasts, mesothelial cell, smooth muscle cells, adipocytes, immune cells, and neurons) and their individual, disease-specific gene expression profile (65). The authors identified gene regulatory networks and disease driver candidates by intersection of the SNS data sets with the disease risk GWAS data (65). This data extended descriptive cellular characterization of the human heart (3, 41) toward understanding the precise underlying disease progression mechanisms.

Altogether, these recent discoveries serve as a blueprint for how knowledge about gene expression dynamics, collected from single cell responses under physiological and pathological conditions, provides unprecedented information. Invaluable insight that supports context-specific therapeutic approaches based on transcriptional modulation is the observation that altered SC transcriptomic profiles in the human diseased hearts seem to reverse toward normal state with improved organ function (3). This indicates that transcriptional profile and function are coupled. Furthermore, as abovementioned, the information collected from current studies, such as HIF1α and ZEB2 expression promoting cardiomyocytes proliferation endogenously, can be used to transiently boost clusters of cardiomyocytes toward a more regenerative state in ischemic conditions. Based on the data that ACKR1+ endothelial cell transplantation preserves cardiac function upon ischemia, an approach using suitable adeno-associated virus (AAV) serotype or other non-viral vector delivery approach can be designed for enhancing *Ackr1* expression in this cell population. This is particularly motivating toward developing more specific strategies that can restore homeostatic cell states for a wide number of human cardiovascular pathologies ranging from adaptive cardiac remodeling to heart failure. The next logical step is to actively interfere with the identified aberrant gene expression and to rewire gene programs for the prevention of heart failure progression. In this context, synthetic control of transcription to restore endogenous homeostatic transcriptional programs of the heart specifically in the desired cell-types offers a suitable platform. In terms of budget, current advances of protocols as well as standardization of bioinformatics pipelines warrants the SCS approach as a realistic option for clinical applications in the near future. Importantly, conventional single-cell RNA-seq analysis may not be sufficient for obtaining the information necessary for a deeper understanding of molecular behavior and therefore combined bulk sequencing analysis will be mandatory for a more precise analysis (66).

## Adapting Synthetic Control of Transcription to the Heart

Regulation of gene expression relies on transcription factors availability and their activity, as well as on chromatin state and nucleosome positioning that determines RNA polymerases recruitment to a specific gene locus (67). This complex process has turned transcriptional control “undruggable” for many years (67). Recent approaches tackling this issue deploy epigenetic modifiers and synthetic transcription factors driven by DNA binding element systems such as engineered zinc finger, transcriptional activator-like elements (TALEs) or aim at repurposing programmable CRISPR/Cas9 systems (68–70). Beyond genome editing activity of the CRISPR system, epigenomic modifications can now be achieved at a specific genomic locus by using mutated catalytically inactive dead (d) Cas9 protein fused to effector domains (68). This works by carefully choosing where guide (g) RNA molecules bind relative to transcriptional start sites (TSS) in the genome. This allows for recruitment of dCas9 with activator or inhibitor domains to a specific locus of interest. Consequently, chromatin landscape modification or further recruitment of factors that lead to tailored transcriptional modulation is possible. This is extensively reviewed elsewhere (67, 71, 72). Hence, the limitation of strict control over endogenous gene expression *in vivo* that has long been a tedious work for researchers is now alleviated by the use of the RNA-guided programmable endonuclease systems associated with transcriptional modifiers (67). This offers the ability to precisely modify endogenous gene expression to program cell and tissue behavior (67). Thus, all the efforts that have been conducted for decades in order to understand how exactly transcriptomic changes cause disease conditions can now be exploited to develop therapeutic concepts by applying rapidly evolving CRISPR/Cas9 technologies.

CRISPR/Cas9 technology is rapidly advancing in the medical world with the development of therapies for blood disorders, Duchenne muscular dystrophy, cystic fibrosis, and cancer (73–75). Yet, the CRISPR-mediated transient transcriptional activation or repression of genes, desirable when considering changing the course of complex diseases such as metabolic diseases or tissue regeneration (76), is still in its infancy. Endogenous regulatory mechanisms of genome function as well as issues concerning CRISPR-mediated transcriptional engineering need to be addressed before this technology reaches use in the clinic, and is discussed elsewhere (67). Initial generation of dCas9-based transcriptional modulation platforms consisted of transcriptional activators derived from herpes simplex virus, VP16. Second-generation systems resulted from combination of bi/or tripartite activators such as VP64, VPR, SAM, the peptide scaffold-based activator SunTag-VP64 and RNA containing aptamers with increased activation efficiency (67, 72). The same is true for synthetic repression, where the initial KRAB repressor domain has been improved by several bipartite repressors consisting of KRAB and a secondary repressor domain (ZIM3, KOX1, MeCP2, DNMT3A, DNMT3L) (72, 77, 78). All these systems allow to fine-tune the intensity of gene modulation according to the biological needs and represent promising tools to modulate the cellular epigenome. Importantly, titration of gene activity is possible with the development of advanced gene activator platforms by expanding homomeric (79) or by using heteromeric transactivation domains (80). Additionally, the selection of gRNA target sites upstream of the TSS was sufficient to modulate drug resistance phenotypes according to expression levels indicating fine-tuning of gene activity to biologically relevant levels (79). Furthermore, tiling of gRNAs in the TSS upstream region was consistently reported as an option to adjust gene activation strength (81–83). Altogether, Cas9-transcription factor characteristics and careful gRNA selection are therefore suitable for unprecedented control of endogenous gene activity, an advantage over classical cDNA delivery via AAV which will be further discussed below.

## CRISPR-Mediated Control of Transcription in Preclinical Models

CRISPR-based synthetic transcriptional control may lay the basis for personalized and precision medicine. Efficient transcription regulation mediated by CRISPR-mediated gene activation (CRISPRa) systems was demonstrated *in vivo* in the brain, liver, kidney and skeletal muscle as well as mouse model of human diseases including muscle dystrophy, diabetes, kidney and brain diseases using different delivery methods (84–87). Preclinical models using dCas9-targeted transcription factor regulation have shown great promise for treating disorders such as Duchenne's muscular dystrophy, type 1 diabetes, acute kidney disease and retinitis pigmentosa (84, 85, 88). Liao at al. developed a mouse model, in which transcriptional activators were separated from constitutively expressed Cas9 (active or inactive, Cas9a and Cas9i, respectively) (85). This consisted of a combination including gRNAs engineered to contain two MS2 domains for recruiting the MS2:P65:HSF1 (MPH) transcriptional activation complex to the target locus, which was introduced with an AAV serotype 9. MS2 binds a specific stem-loop structure allowing assembly of RNA-protein complexes and it is used as tagging technique (89). Using this system, they showed amelioration of acute kidney injury by induced expression of the protective protein *Klotho* or the anti-inflammatory IL-10. Next, they triggered trans-differentiation of liver cells into insulin-producing cells by induction of pancreatic and duodenal homeobox gene 1 (*Pdx1*) in liver cells; which improved hyperglycemia in streptozotocin (STZ)-induced diabetes model via tail vein injection of AAV-gRNAs. Moreover, they showed that transcriptional induction of *utrophin*, a protein product which is very similar to dystrophin, improved muscle strength in a mouse model of Duchenne muscular dystrophy (DMD) by local application of the AAV-gRNAs (85). Additionally, a kidney-specific epigenetic modifier with dCas9TET3 fusion proteins to induce gene activity was shown to be efficient by Xu et al. (90). A double transgenic mouse was generated in a *Sim1* heterozygous background, which normally develops obesity. The second transgene consists of a dCas9 fused to a transcriptional activator VP64 as well as a sgRNA targeting the *Sim1* promoter or enhancer (88). By using this system to activate *Sim1* expression from the healthy intact allele, the obesity phenotype was rescued upon targeting of both, *Sim1* promoter or enhancer. The same rescue was observed upon direct delivery of three different AAV particles carrying the dCas9VP16, the gRNA-promoter *Sim1* and the gRNA-enhancer *Sim1* into the hypothalamus. These examples elegantly demonstrate that CRISPR-based transcriptional modulation can also be applied for epigenetically-mediated correction of a genetic disease without the need of modifying the mutated gene-coding DNA sequence. Meng et al. engineered bone marrow derived mesenchymal stem cell (MSC) to overexpress *Il-10* using CRISPRa based on dCas9-VP64-MS2 system (91).

IL-10 improved myocardial infarction; which is hampered in patients with diabetes due to MSC dysfunction. Engineered MSCs overexpressing *Il-10* were transplanted in a diabetic mice model with myocardial infarction, which substantially suppressed inflammation, improved cardiac functional recovery, alleviated cardiac injury, decreased apoptosis of cardiac cells, and increased angiogenesis (91). Other studies showed that CRISPRa approaches can decrease seizures and rescue cognitive deficits in a rodent model of epilepsy as well as demonstrated the utility of CRISPRa system for *in vivo* screening, e.g., in liver or brain (86, 87). Common efforts from E. Olson's and L. Zelarayán's Labs resulted in the establishment of a mouse model for cardiomyocyte-specific, CRISPR-mediated transcriptional modulation. The system is based on the constitutive expression of dCas9VPR combined with systemic administration of gRNA driving dCas9 to specific loci via AAV serotype 9, which showed robust, safe and specific single or multiplex activation of targeted genes (92). This model represents a rapid and powerful technical platform for gene activation in postnatal cardiomyocytes in preclinical proof-of-concepts. All of these studies demonstrate the feasibility of CRISPR-based methods for transcriptome modulation and set the stage for future optimization in both, basic and clinical research.

## CRISPR-Mediated Control of Transcription to Enhance Cardiac Regeneration

**Figure 1:**
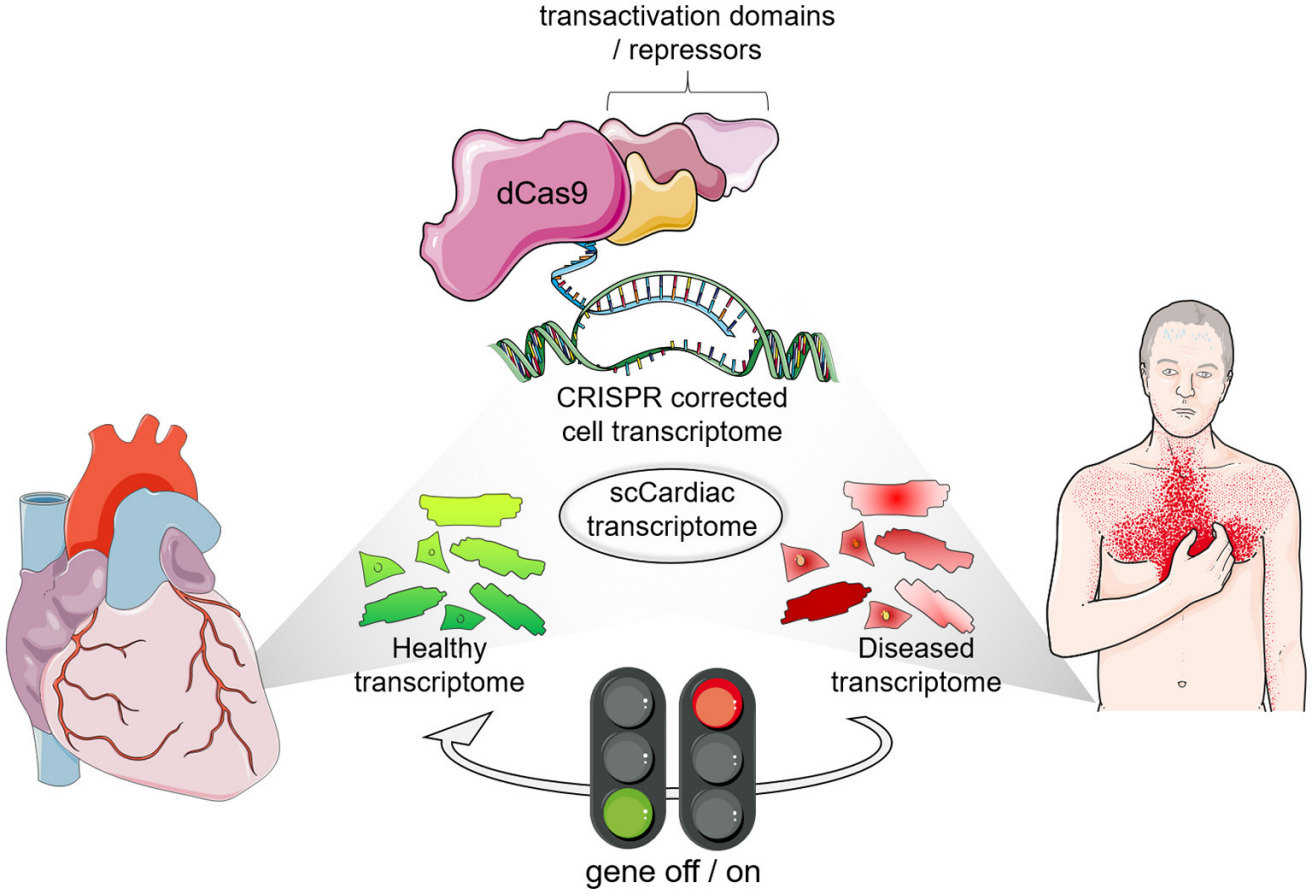
The era of transcription profile analyses and transcriptional engineering at single cell resolution. Single cell (SC) transcriptomics identified cell types in healthy and diseased cardiomyocytes along with their transcriptional profile. Interference with gene expression is possible with CRISPR-based synthetic transcription factors to steer gene expression profiles of specific subpopulations of target cells in the heart.

Although modulating individual factors in adult cardiomyocytes did enable some proliferative activity (93, 94), overexpression of a combination of cell cycle regulators increased the effect on cardiomyocyte proliferation and improved cardiac function after ischemic injury (95). Moreover, as aforementioned, effective cardiomyocytes' regeneration requires coordinated structural and metabolic alteration, which demands the targeting of multiple mechanisms. A highly attractive feature of the CRISPR-based technology is the possibility of simultaneous manipulation of multiple genes that can be exploited to efficiently induce cardiomyocytes renewal. The notion that a disease phenotype is triggered by dysregulation of several factors affecting one or more networks supports the use of multiple genes manipulation targeting dynamic gene networks, which perturbation results in a disease phenotype. In this context, CRISPR-associated RNA scaffolds were shown to provide a powerful way to construct not only multiple, but also orthogonal synthetic gene expression programs (96, 97). Such system was applied to modulate a branched metabolic pathway in yeast, in which multiplexed transcription activation and repression is carried out using distinct single gRNAs modified with RNA aptamers, termed scaffold RNAs. These aptamers can recruit either binding protein fused to a Krüppel-associated box (KRAB) domain for transcription repression or the MS2 coat protein (MCP) fused to VP64 for transcription activation (97). Another strategy made use of different dCas9 orthologs in a dual inducible and repressible systems for precise and dynamic control of CRISPR-dCas9- and 12a-mediated epigenetic editing tested in HEK293T cells (98). These studies provide promising evidence of the ability to use CRISPR-mediated gene modulation for modeling complex gene networks and reversing a disease condition using orthogonal systems for parallel activation and repression in the same cell. The combination of single cell transcriptomics and the bioinformatic assessment of network activities will provide the information for tailored CRISPR-based synthetic control of transcription. This will allow steering gene expression profiles in order to detour a cell toward a physiological state and prevent organ deterioration in a multifactorial and disease-specific manner (Figure 1).

Besides targeting cardiomyocytes, which depending on the disease condition and extent of the damage may not be efficient, a promising approach to promote regeneration of heart tissue is to convert resident non-cardiomyocytes cells directly into *de novo* cardiomyocytes (10). Direct reprogramming *in vivo* has been reported in mice by using GATA4, MEF2C, and TBX5 (GMT) or GMT factors plus HAND2 (GHMT) reprogramming cocktails with retroviral delivery in order to infect proliferating cells such as activated cardiac fibroblasts after myocardial injury (99, 100). This approach generated new cardiomyocyte-like cells from activated cardiac fibroblasts. However, direct reprogramming showed relatively low reprogramming efficiency (10). Adding of ZNF281 to the reprogramming cocktail repressed genes associated with the inflammatory response as well as regulated cardiac gene expression by interacting with the transcription factor GATA4 (101). Additional factors improving cardiac reprogramming efficiency include the modification of endogenous signaling pathways such as RAC-α serine/threonine-protein kinase (AKT1), transforming growth factor-β (TGFβ), WNT, and Notch signaling (10, 102, 103). Furthermore, enhanced cardiac reprogramming was observed by suppressing the expression of the Polycomb complex protein BMI1 and the splicing factor polypyrimidine tract-binding protein 1 (PTB), while indicating the repressive role of these factors for cardiac reprogramming (104, 105). Trans-differentiation induced by activating endogenous gene expression with the use of the CRISPR-dCas9 system has been reported in different cell lines including neonatal mouse fibroblasts (10, 85, 106–108). However, it was shown that endogenous cardiac transcription factor activation is necessary for expression of maturation genes but not sufficient to induce efficient cardiac fibroblast trans-differentiation (108). It will be interesting to evaluate whether combinatorial activation and/or repression, allowing for more precise control of multiple pathways in orthogonal directions, could enhance cardiomyocytes reprogramming efficiencies by harnessing knowledge about the epigenetic landscape and modulating factors of cardiomyogenic cells (109).

**Figure 2:**
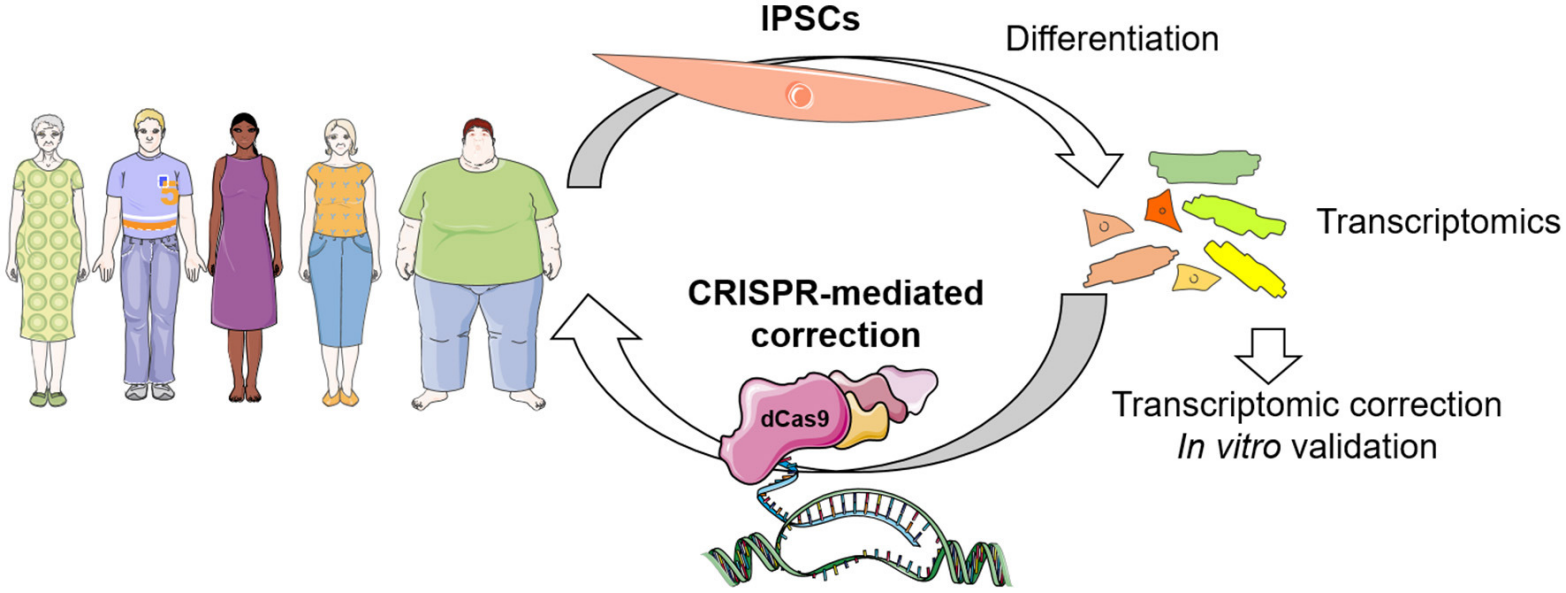
Envisioned concept of CRISPR-dCas9 system in patient-specific therapeutics. Identification of transcriptional networks dysregulation can be achieved in patient specific iPSC-derived cells, which can be corrected by CRISPR-mediated gene modulation *in vitro* for validation, and finally *in vivo* for personalized therapeutics in the near future.

A further application of CRISPR-dCas9 systems will include patient-specific induced pluripotent stem cells (iPSC) for derivation of specific cell types for transcriptomic approaches. The iPSCs offer an attractive experimental platform, paving the way for the development of personalized medicine in cardiovascular diseases (110). CRISPR/dCas9 activation and interference systems were widely used for genome-scale screening (96, 111–113). Upon identification of transcriptional networks dysregulation in patient specific iPSC-derived cells, the amalgamation of CRISPR-mediated gene modulation with iPSC technology may allow reverting disease condition in a dish as a basis to translate the personalized approach to the patient without affecting the genomic DNA. This will include the delivery of a CRISPR-gene modulation molecular tool that will restore the altered transcriptome in specific cells for precise (patient and disease specific) therapeutical applications. With these improvements, personalized medicine could be a reality for many patients, minimizing side effects (Figure 2).

The concept of using single cell (SC) transcriptome for personalized medicine is already an important research focus for the investigation and treatment of multifactorial diseases. Recently, a pan-European initiative (“Life Time”: Revolutionizing Healthcare by Tracking and Understanding Human Cells during Disease) as well as national initiatives (e.g., Berlin Cell Hospital and Virchow 2.0, Germany) have been initiated. These and similar consortia aim at targeting human cells during the onset and progression of complex diseases as well as at analyzing their response to therapy at single-cell resolution (114). Integration of large molecular and clinical datasets will identify molecular mechanisms and create predictive computational models of disease progression allowing the implementation of gene or pathway-directed targeted therapy (114, 115). SCS technology has already contributed to the identification of novel disease biomarkers helping in the diagnosis and refinement of treatments. High-resolution SC transcriptomics will be vital in dissecting how these new treatments affect cell populations receiving the cell precision therapies (115). The CRISPR toolbox is an emerging opportunity to therapeutically modulate cellular states by the use of gene or base editing and synthetic transcription. CRISPR/Cas9-based gene editing approaches for prevention of cardiovascular disease have been demonstrated (71, 116). CRISPR base editors that are delivered *in vivo* using lipid nanoparticles were shown to efficiently and precisely modify disease-related genes in living cynomolgus monkeys (117). In this study, *PCSK9*, a well-established target in atherosclerosis, was mutated *in vivo* using CRISPR base editors leading to a variant that resulted in lower levels of low-density lipoprotein (LDL) cholesterol in the blood and reduced the risk of atherosclerotic cardiovascular disease (117). In the same manner, identification of specific targets by omics approaches will allow to combine with CRISPR-based modulation approaches in order to target a specific (genomic or epigenomic) perturbation in a disease and patient-specific manner. Logistically, primary patient tissue or iPSC cells, differentiated into a desired cell type, can be used for extracting omics information to be further applied in pathway analysis and network perturbation identification. This would lead to deciphering the set of factors that may need to be modulated for reverting a disease condition, which will be tested experimentally *in vitro* before ultimately reaching the patient. Technical challenges that need to be overcome include standardization of the methods, costs and user-friendly analysis tools.

## Advantages and Challenges of CRISPR-Mediated Transcriptional Control

While the CRISPR/Cas9 system has demonstrated a great promise for a variety of applications, there are several factors that influence its efficacy as well as its safety which must be addressed, especially when the goal is *in vivo* human gene therapy. Last but not least, there are ethical implications that need to be carefully considered, and are discussed extensively elsewhere (118–120). A more worrisome problem includes specific biosafety regulatory challenges and ethical issues concerning applications of CRISPR technology for irreversibly editing the human genome. Nevertheless, ethical concerns need to be addressed properly, which may not be unique when considering other interventions that influence human biology (119).

In respect to tool design, the necessary factors that require careful examination include target DNA site selection, sgRNA design, off-target effects and the method of delivery, the latter representing a major obstacle for use of CRISPR-based editors for *in vivo* applications (121). A powerful advantage of the CRISPR/Cas9 system is the ability to especifically target any 23-bp sequence that contains a PAM motif on either strand of DNA (121). However, single and multiple-base mismatches can be tolerated specially at greater distances from the PAM resulting in off-target effects (122–129). Importantly, the catalytically inactive Cas9 leaves the genome unaffected, significantly reducing the concerns over off-target effects (92, 130). Therefore, lower risk of side-effects are introduced by using a dCas9; however, this needs to be addressed on individual sgRNAs and in a context-specific manner. In order to reduce off-target events, rational design of the sgRNA has been the subject of a significant body of work resulting in many criteria and no simple rules (121). Comparing predictions from several sgRNA design tools with experimental results published in SpyCas9 off-target studies, showed evidence of algorithmic overfitting (124). They indicated the importance of using a model trained on data from the same gRNA expression system, which are currently few, especially for tissues when *in vivo* experiments are deployed.

From the clinical perspective, CRISPR-mediated control of gene expression offers several advantages compared to previous methods based on the expression of an open reading frame of the gene of interest, lacking physiologically relevant splice variants with exogenous, and thus uncontrolled expression. CRISPR-mediated regulation has overcome these obstacles and can now generate unprecedented levels of endogenous control while simultaneously offering a multiplexing possibility (131). The major challenge of CRISPR-based therapies is the delivery. Delivery systems for the CRISPR machinery can be classified into three general groups: physical delivery, viral vectors, and non-viral vectors. Viral delivery vectors include specifically engineered AAV, and full-sized adenovirus and lentivirus vehicles. These are the most common CRISPR/Cas9 delivery methods for *in vivo* approaches (121). AAV is considered a suitable vehicle for gene therapy since it is not known to cause any pathologies in humans, and there is a wide range of serotypes allowing for infection of a multitude of cells with different specificities. Moreover, the virus itself is able to efficiently transduce cells, while provoking little to no innate or adaptive immune response or associated toxicity, at least upon the first treatment with a certain serotype (121, 132). Thus, due to their well-proven safety profiles, AAVs are currently the best choice for nucleic acid-based therapy in clinical trials. AAVs, however, have the disadvantage of a small payload of ~4.7 kb, which can become a limitation considering all the necessary components of the CRISPR activation or repression systems (76, 133). Several approaches are under development to circumvent these hurdles, including the profiling of non-viral nanoparticles for gene delivery and decreasing the size of individual Cas9 components (134). Successful packaging of the SpyCas9 and sgRNA into two separate AAV particles and using them for co-transduction has been already reported (135). On one hand, this increases the overall size of the constructs that can be used. On the other hand, this naturally adds more complexities than those existing with a single vector (121). A further approach includes a split Cas9 system, in which the Cas9 C-terminal region is packaged into one AAV vector and the Cas9 N-terminus is packaged into a second AAV vector (136, 137). Reconstitution of the two Cas9 halves results in a functional Cas9 with editing efficiency comparable to the native Cas9, allowing for the use of larger overall Cas9 variants with AAV particles. This has also been proven to be effective in gene editing in pig and human models of Duchenne muscular dystrophy (116, 138). Moreover, the identification of a small Cas9 ortholog from Staphylococcus auricularis (SauriCas9) that can be packaged into an AAV for genome editing, has broadened the possibilities of efficient delivery and can be adapted for gene modulation, further expanding the CRISPR toolbox for epigenetic regulation (139). Non-viral delivery of Cas9 for genome editing have been demonstrated less efficient than viral methods, however, they could allow repeated dosing by using e.g., lipid-based nanoparticles (140). Other delivery strategies have been applied *in vivo*, including direct mRNA delivery and ribonucleoprotein (RNP) delivery with lipid nanoparticles (LNP), especially for genome editing (140). Lipofectamine has been used to deliver base editors to the murine ear, however, entailing toxicity, which promoted the development of more biocompatible lipid formulations that can be used to deliver the Cas9 RNP *in vivo*. These formulations include gold nanowires, gold nanoclusters, black phosphorus nanosheets and nanoscale zeolitic imidazole frameworks (ZIFs). *In vivo* efficacies of these delivery systems are yet to be determined. Thus, there is a growing need for the next-generation more efficient vectors to be developed (141–144).

Despite these hurdles, CRISPR/Cas9-based therapies have begun their path into the clinic. CRISPR-based gene editing clinical trials for sickle cell disease and beta-thalassemia (CTX001) have paved the way for CRISPR-mediated therapies and further optimizations (145). This has been followed by AAV-based clinical trials and planned non-viral nanoparticle-based delivery of CRISPR to the liver (NTLA-2001) (145). Future studies are necessary to determine pre-existing immunity against candidate Cas9 proteins in humans. Also, the combination of cell- and tissue-specific regulatory components with broad tropism AAV vectors will help to fine-tune the localization of the effector components, while providing increased specificity and safety (76). Many classical targets considered “undruggable” came into play with expression interference strategies such as siRNA (146) and proteolysis-tags technologies (147). With CRISPR/Cas9 gene activity modulation, a powerful approach to precisely target candidate expression mechanisms at the transcriptional level emerges, further expanding our targeting scope. While enzyme activation with classical pharmacological approaches such as small molecules is limited to a small fraction of candidate targets (148), CRISPR gene modulation harbors the potential for gain-of-function mode of actions including transcription factors [i.e., Pdx1 (85) and c-myc (86)], formerly deemed difficult-to-drug (149). Furthermore, CRISPR gene modulation was shown to be efficient for congenital diseases based on haploinsufficiency and diseases caused by loss of a gene product in animal models. An endogenous gene product can be normalized from the healthy allele [as shown for *Sim1* haploinsufficiency in obesity (88)] or replaced by a similar transcript [as shown for *utrophin*, replacing the lack of dystrophin for Duchenne muscular dystrophy (85)]. These therapies present challenges when using pharmacological applications.

Simultaneous activation and repression of multiple genes leading to network modulation rather than unidirectional regulation may be of more therapeutic relevance. With the intensified investigation of endogenous gene regulatory networks in the SC-specific context, the gene network engineering via CRISPR systems is highly attractive for the reestablishment of homoeostatic gene regulatory networks upon disease conditions. Such a high precision tuning of the defined sets of synergistic genes will result in the extraordinary control over cell behavior (131), allowing the induction of tailored reparative responses using the own cell machinery in the mature organ. While they can be exploited to enhance regenerative processes of cells, tissues, and organs, these advances need further technological development along with a better understanding of how exactly epigenomic and transcriptomic changes mediate phenotypic effects at the single cell resolution (67). This will allow for more precisely targeted approaches adjusted to the physiological needs.

## Controlling Cas9 Function and Side-Effects

To restrict Cas9 activity, and thus reduce the off-target effects, attempts for temporally restricted (d)Cas9 expression were developed including chemical and light controlled gene activity modulation. Detailed reviews regarding inducible Cas9 systems discussed background (leaky) activity, editing effectivity, and reversibility for gene editing approaches (150, 151). We therefore summarize inducible systems specifically adapted for endogenous gene activity modulation here. Temporal control was harnessed by decoupling Cas9 from transcriptional modulators with conditional chemical or light induced assembly of the synthetic transcription factor. Tested chemical and light inducible elements included: (1) Absicic acid (ABI-PYL1), Giberrellin (GIB1-GAI), and Rapamycin (FKBP-FRB) systems as well as (2) red-light (PHYB-PIF), and blue-light (CRY2PHR-CIBN) inducible systems (98, 152, 153).

Small molecule based Cas9-DNA interference was demonstrated and applicable for CRISPRa approaches reducing gene expression of up to 89% (154). While effectively limiting (d)Cas9 activity, these systems rely on constitutive expression and presence of (d)Cas9 and effector domain proteins harboring potential for unwanted cellular and organismal effects. To overcome this, Trimetoprim or Doxycycline responsive promoter elements driving dCas9 transcription were successfully tested for induced dCas9 expression with concomitant (multiplexed) gene activation (155–157). Additionally, degron motif-based “suicide-tags” for protease inhibition-dependent Cas9 expression was presented as a promising option for Cas9 activity restriction (158). Taming Cas9 expression or activity using pharmacological approaches might therefore be a prospective route for temporally restricted or pulsed endogenous gene activation, possibly reducing expected immune responses upon constitutive Cas9 expression (159) and deserves a future validation in *in vivo* models. Bioengineering of Cas9 proteins as well as elaborate cell-type specific and temporally resolved Cas9 expression systems (151) are therefore essential ventures for safe and limited CRISPR/Cas9 based applications (71). Protein-based anti-CRISPRs, which are accessory proteins with fewer than 200 amino acids called “anti-CRISPRs,” can function as antagonists of CRISPR systems and achieve context-specific inhibition of Cas9. This will offer a solution for mitigating the problem of off-target cleavage as well as for limiting Cas9 activity on the genome (160). To what extent these approaches can be transferred to the clinic is still uncertain, nevertheless they warrant further studies.

## Conclusion

Research efforts during the past decades have broadened our insights into the molecular determinants of cardiac disease. With the recent emergence of the SCS multi-omics profiling, more detailed and comprehensive understanding of the basic molecular profiles of disease-associated perturbations within each cell in the human heart could be achieved, fine-tuning our previous knowledge. Combining this information with the revolutionizing CRISPR technology will enormously advance medical research and open a new chapter of precision and personalized medicine. Within the last few years of research, CRISPR-mediated gene editing has already entered the clinic, making the application of further CRISPR approaches for synthetic transcription a realistic option for more specific treatments of different cardiac disease entities. Furthermore, integration of patient-specific data and human-based *in vitro* models, will help to identify more personalized therapies for rare diseases. Such an approach will necessarily include collection of biological information from a patient, integration of a suitable iPSC-model to test patient- and/or disease-specific synergistic network modulation and their safety, and ultimately fine-tuning of a precise therapeutic approach. All these tools will broaden our understanding of human- and disease-specific effects as well as provide us with information about safety of proposed modulations. With these advances, a reshaped personalized management of complex human diseases will become a realistic approach. Reduction of costs for development and production of such approaches is mandatory to allow disease-specific and individualized therapies in the future. Joined efforts of clinicians, basic researchers and industry partners is already now facilitating rapid advancements of the discussed technologies that will dramatically impact biomedical research and disease treatments in the future.

## Author Contributions

ES, SL, DP, and LZ conceptualized, wrote, and revised the manuscript and figures. All authors contributed to the article and approved the submitted version.

## Funding

The authors are supported by the DFG grant SFB1002 C07 and INF; the H2020-EU CRYSTAL3 to LZ. The Helmholtz Association and the ERC-RA-0047 to DP as well as the DZHK (German Center for Cardiovascular Research) to ES, DP, and LZ.

## Conflict of Interest

The authors declare that the research was conducted in the absence of any commercial or financial relationships that could be construed as a potential conflict of interest.

## Publisher's Note

All claims expressed in this article are solely those of the authors and do not necessarily represent those of their affiliated organizations, or those of the publisher, the editors and the reviewers. Any product that may be evaluated in this article, or claim that may be made by its manufacturer, is not guaranteed or endorsed by the publisher.

## References

[1] WHO. Cardiovascular Diseases. World Health Organization (2017).

[2] Riehle C Bauersachs J. Small animal models of heart failure. Cardiovasc Res. (2019) 115:1838–49. 10.1093/cvr/cvz161 31243437 PMC6803815

[3] Wang L Yu P Zhou B Song J Li Z Zhang M. Single-cell reconstruction of the adult human heart during heart failure and recovery reveals the cellular landscape underlying cardiac function. Nat Cell Biol. (2020) 22:108–19. 10.1038/s41556-019-0446-7 31915373

[4] Kloner RA Brown DA Csete M Dai W Downey JM Gottlieb RA. New and revisited approaches to preserving the reperfused myocardium. Nat Rev Cardiol. (2017) 14:679–93. 10.1038/nrcardio.2017.102 28748958 PMC5991096

[5] Leong YY Ng WH Ellison-Hughes GM Tan JJ. Cardiac stem cells for myocardial regeneration: they are not alone. Front Cardiovasc Med. (2017) 4:47. 10.3389/fcvm.2017.00047 28770214 PMC5511846

[6] Vujic A Natarajan N Lee RT. Molecular mechanisms of heart regeneration. Semin Cell Dev Biol. (2019) 100:20–8. 10.1016/j.semcdb.2019.09.003 31587963 PMC7071978

[7] Bongiovanni C Sacchi F Da Pra S Pantano E Miano C Morelli MB. Reawakening the intrinsic cardiac regenerative potential: molecular strategies to boost dedifferentiation and proliferation of endogenous cardiomyocytes. Front Cardiovasc Med. (2021) 8. 10.3389/fcvm.2021.750604 34692797 PMC8531484

[8] Bergmann O Bhardwaj RD Bernard S Zdunek S Barnabe-Heider F Walsh S. Evidence for cardiomyocyte renewal in humans. Science. (2009) 324:98–102. 10.1126/science.1164680 19342590 PMC2991140

[9] Senyo SE Steinhauser ML Pizzimenti CL Yang VK Cai L Wang M. Mammalian heart renewal by pre-existing cardiomyocytes. Nature. (2013) 493:433–6. 10.1038/nature11682 23222518 PMC3548046

[10] Hashimoto H Olson EN Bassel-Duby R. Therapeutic approaches for cardiac regeneration and repair. Nat Rev Cardiol. (2018) 15:585–600. 10.1038/s41569-018-0036-6 29872165 PMC6241533

[11] Agah R Kirshenbaum LA Abdellatif M Truong LD Chakraborty S Michael LH. Adenoviral delivery of E2F-1 directs cell cycle reentry and p53-independent apoptosis in postmitotic adult myocardium *in vivo*. J Clin Invest. (1997) 100:2722–8. 10.1172/JCI119817 9389735 PMC508475

[12] Alam P Haile B Arif M Pandey R Rokvic M Nieman M. Inhibition of senescence-associated genes Rb1 and Meis2 in adult cardiomyocytes results in cell cycle reentry and cardiac repair post-myocardial infarction. J Am Heart Assoc. (2019) 8:e012089. 10.1161/JAHA.119.012089 31315484 PMC6761626

[13] Heallen T Morikawa Y Leach J Tao G Willerson JT Johnson RL. Hippo signaling impedes adult heart regeneration. Development. (2013) 140:4683–90. 10.1242/dev.102798 24255096 PMC3833428

[14] Izumi M Fujio Y Kunisada K Negoro S Tone E Funamoto M. Bone morphogenetic protein-2 inhibits serum deprivation-induced apoptosis of neonatal cardiac myocytes through activation of the Smad1 pathway. J Biol Chem. (2001) 276:31133–41. 10.1074/jbc.M101463200 11408477

[15] Kirshenbaum LA Abdellatif M Chakraborty S Schneider MD. Human E2F-1 reactivates cell cycle progression in ventricular myocytes and represses cardiac gene transcription. Dev Biol. (1996) 179:402–11. 10.1006/dbio.1996.0270 8903355

[16] Kusano KF Pola R Murayama T Curry C Kawamoto A Iwakura A. Sonic hedgehog myocardial gene therapy: tissue repair through transient reconstitution of embryonic signaling. Nat Med. (2005) 11:1197–204. 10.1038/nm1313 16244652

[17] Leach JP Heallen T Zhang M Rahmani M Morikawa Y Hill MC. Hippo pathway deficiency reverses systolic heart failure after infarction. Nature. (2017) 550:260–4. 10.1038/nature24045 28976966 PMC5729743

[18] Mahmoud AI Kocabas F Muralidhar SA Kimura W Koura AS Thet S. Meis1 regulates postnatal cardiomyocyte cell cycle arrest. Nature. (2013) 497:249–53. 10.1038/nature12054 23594737 PMC4159712

[19] Ogawa M Geng FS Humphreys DT Kristianto E Sheng DZ Hui SP. Kruppel-like factor 1 is a core cardiomyogenic trigger in zebrafish. Science. (2021) 372:201–5. 10.1126/science.abe2762 33833125

[20] Porrello ER Mahmoud AI Simpson E Hill JA Richardson JA Olson EN. Transient regenerative potential of the neonatal mouse heart. Science. (2011) 331:1078–80. 10.1126/science.1200708 21350179 PMC3099478

[21] Singh BN Koyano-Nakagawa N Gong W Moskowitz IP Weaver CV Braunlin E. A conserved HH-Gli1-Mycn network regulates heart regeneration from newt to human. Nat Commun. (2018) 9:4237. 10.1038/s41467-018-06617-z 30315164 PMC6185975

[22] Tao G Kahr PC Morikawa Y Zhang M Rahmani M Heallen TR. Pitx2 promotes heart repair by activating the antioxidant response after cardiac injury. Nature. (2016) 534:119–23. 10.1038/nature17959 27251288 PMC4999251

[23] Xin M Kim Y Sutherland LB Murakami M Qi X McAnally J. Hippo pathway effector Yap promotes cardiac regeneration. Proc Natl Acad Sci USA. (2013) 110:13839–44. 10.1073/pnas.1313192110 23918388 PMC3752208

[24] Yu W Huang X Tian X Zhang H He L Wang Y. GATA4 regulates Fgf16 to promote heart repair after injury. Development. (2016) 143:936–49. 10.1242/dev.130971 26893347

[25] Zhang D Wang Y Lu P Wang P Yuan X Yan J. Author Correction: REST regulates the cell cycle for cardiac development and regeneration. Nat Commun. (2018) 9:167. 10.1038/s41467-017-02617-7 29330540 PMC5766539

[26] Quaife-Ryan GA Sim CB Ziemann M Kaspi A Rafehi H Ramialison M. Multicellular transcriptional analysis of mammalian heart regeneration. Circulation. (2017) 136:1123–39. 10.1161/CIRCULATIONAHA.117.028252 28733351 PMC5598916

[27] Chen J Huang ZP Seok HY Ding J Kataoka M Zhang Z. mir-17-92 cluster is required for and sufficient to induce cardiomyocyte proliferation in postnatal and adult hearts. Circ Res. (2013) 112:1557–66. 10.1161/CIRCRESAHA.112.300658 23575307 PMC3756475

[28] Eulalio A Mano M Dal Ferro M Zentilin L Sinagra G Zacchigna S. Functional screening identifies miRNAs inducing cardiac regeneration. Nature. (2012) 492:376–81. 10.1038/nature11739 23222520

[29] Gong R Wang X Li H Liu S Jiang Z Zhao Y. Loss of m6A methyltransferase METTL3 promotes heart regeneration and repair after myocardial injury. Pharmacol Res. (2021) 105845. 10.1016/j.phrs.2021.105845 34428587

[30] Tian Y Liu Y Wang T Zhou N Kong J Chen L. A microRNA-Hippo pathway that promotes cardiomyocyte proliferation and cardiac regeneration in mice. Sci Transl Med. (2015) 7:279ra238. 10.1126/scitranslmed.3010841 25787764 PMC6295313

[31] Chen Y Luttmann FF Schoger E Scholer HR Zelarayan LC Kim KP. Reversible reprogramming of cardiomyocytes to a fetal state drives heart regeneration in mice. Science. (2021) 373:1537–40. 10.1126/science.abg5159 34554778

[32] Bersell K Arab S Haring B Kuhn B. Neuregulin1/ErbB4 signaling induces cardiomyocyte proliferation and repair of heart injury. Cell. (2009) 138:257–70. 10.1016/j.cell.2009.04.060 19632177

[33] D'Uva G Aharonov A Lauriola M Kain D Yahalom-Ronen Y Carvalho S. ERBB2 triggers mammalian heart regeneration by promoting cardiomyocyte dedifferentiation and proliferation. Nat Cell Biol. (2015) 17:627–38. 10.1038/ncb3149 25848746

[34] Honkoop H Bakker DE Alla Aharonov FK Shakked A Nguyen PD Heus C. Single-cell analysis uncovers that metabolic reprogramming by ErbB2 signaling is essential for cardiomyocyte proliferation in the regenerating heart. eLife. (2019) 8:e50163. 10.7554/eLife.50163 31868166 PMC7000220

[35] Cahill TJ Choudhury RP Riley PR. Heart regeneration and repair after myocardial infarction: translational opportunities for novel therapeutics. Nat Rev Drug Discov. (2017) 16:699–717. 10.1038/nrd.2017.106 28729726

[36] Galdos FX Guo Y Paige SL VanDusen NJ Wu SM Pu WT. Cardiac regeneration: lessons from development. Circ Res. (2017) 120:941–59. 10.1161/CIRCRESAHA.116.309040 28302741 PMC5358810

[37] Gemberling M Karra R Dickson AL Poss KD. Nrg1 is an injury-induced cardiomyocyte mitogen for the endogenous heart regeneration program in zebrafish. Elife. (2015) 4. 10.7554/eLife.05871.015 25830562 PMC4379493

[38] Ellen Kreipke R Wang Y Miklas JW Mathieu J Ruohola-Baker H. Metabolic remodeling in early development and cardiomyocyte maturation. Semin Cell Dev Biol. (2016) 52:84–92. 10.1016/j.semcdb.2016.02.004 26912118 PMC4820352

[39] Malek Mohammadi M Abouissa A Azizah I Xie Y Cordero J Shirvani A. Induction of cardiomyocyte proliferation and angiogenesis protects neonatal mice from pressure overload-associated maladaptation. JCI insight. (2019) 5:e128336. 10.1172/jci.insight.128336 31335322 PMC6777810

[40] Sadek H Olson EN. Toward the goal of human heart regeneration. Cell Stem Cell. (2020) 26:7–16. 10.1016/j.stem.2019.12.004 31901252 PMC7257208

[41] Litvinukova M Talavera-Lopez C Maatz H Reichart D Worth CL Lindberg EL. Cells of the adult human heart. Nature. (2020) 588:466–72. 10.1038/s41586-020-2797-4 32971526 PMC7681775

[42] Tombor LS John D Glaser SF Luxan G Forte E Furtado M. Single cell sequencing reveals endothelial plasticity with transient mesenchymal activation after myocardial infarction. Nat Commun. (2021) 12:681. 10.1038/s41467-021-20905-1 33514719 PMC7846794

[43] Efremova M Vento-Tormo M Teichmann SA Vento-Tormo R. CellPhoneDB: inferring cell-cell communication from combined expression of multi-subunit ligand-receptor complexes. Nat Protoc. (2020) 15:1484–506. 10.1038/s41596-020-0292-x 32103204

[44] Doudna JA. The promise and challenge of therapeutic genome editing. Nature. (2020) 578:229–36. 10.1038/s41586-020-1978-5 32051598 PMC8992613

[45] Moorman AF Christoffels VM. Cardiac chamber formation: development, genes, and evolution. Physiol Rev. (2003) 83:1223–67. 10.1152/physrev.00006.2003 14506305

[46] Gladka MM. Single-Cell RNA sequencing of the adult mammalian heart-state-of-the-art and future perspectives. Curr Heart Fail Rep. (2021) 18:64–70. 10.1007/s11897-021-00504-3 33629280 PMC7954708

[47] Yamada S Nomura S. Review of single-cell RNA sequencing in the heart. Int J Mol Sci. (2020) 21:8345. 10.3390/ijms21218345 33172208 PMC7664385

[48] Yekelchyk M Guenther S Preussner J Braun T. Mono- and multi-nucleated ventricular cardiomyocytes constitute a transcriptionally homogenous cell population. Basic Res Cardiol. (2019) 114:36. 10.1007/s00395-019-0744-z 31399804 PMC6689038

[49] Farbehi N Patrick R Dorison A Xaymardan M Janbandhu V Wystub-Lis K. Single-cell expression profiling reveals dynamic flux of cardiac stromal, vascular and immune cells in health and injury. Elife. (2019) 8:e43882. 10.7554/eLife.43882.061 30912746 PMC6459677

[50] Gladka MM Molenaar B de Ruiter H van der Elst S Tsui H Versteeg D. Single-cell sequencing of the healthy and diseased heart reveals cytoskeleton-associated protein 4 as a new modulator of fibroblasts activation. Circulation. (2018) 138:166–80. 10.1161/CIRCULATIONAHA.117.030742 29386203

[51] Foulquier S Daskalopoulos EP Lluri G Hermans KCM Deb A Blankesteijn WM. WNT signaling in cardiac and vascular disease. Pharmacol Rev. (2018) 70:68–141. 10.1124/pr.117.013896 29247129 PMC6040091

[52] Palevski D Levin-Kotler LP Kain D Naftali-Shani N Landa N Ben-Mordechai T. Loss of macrophage Wnt secretion improves remodeling and function after myocardial infarction in mice. J Am Heart Assoc. (2017) 6:e004387. 10.1161/JAHA.116.004387 28062479 PMC5523630

[53] Frey N Richardson JA Olson EN. Calsarcins, a novel family of sarcomeric calcineurin-binding proteins. Proc Natl Acad Sci USA. (2000) 97:14632–7. 10.1073/pnas.260501097 11114196 PMC18970

[54] Kimura W Xiao F Canseco DC Muralidhar S Thet S Zhang HM. Hypoxia fate mapping identifies cycling cardiomyocytes in the adult heart. Nature. (2015) 523:226–30. 10.1038/nature14582 26098368

[55] Gladka MM Kohela A Molenaar B Versteeg D Kooijman L Monshouwer-Kloots J. Cardiomyocytes stimulate angiogenesis after ischemic injury in a ZEB2-dependent manner. Nat Commun. (2021) 12:84. 10.1038/s41467-020-20361-3 33398012 PMC7782784

[56] Krishnan J Suter M Windak R Krebs T Felley A Montessuit C. Activation of a HIF1alpha-PPARgamma axis underlies the integration of glycolytic and lipid anabolic pathways in pathologic cardiac hypertrophy. Cell Metab. (2009) 9:512–24. 10.1016/j.cmet.2009.05.005 19490906

[57] Janbandhu V Tallapragada V Patrick R Li Y Abeygunawardena D Humphreys DT. Hif-1a suppresses ROS-induced proliferation of cardiac fibroblasts following myocardial infarction. Cell Stem Cell. (2021) S1934-5909:00421-5. 10.1016/j.stem.2021.10.009 34762860 PMC9021927

[58] Glembotski CC. The role of the unfolded protein response in the heart. J Mol Cell Cardiol. (2008) 44:453–9. 10.1016/j.yjmcc.2007.10.017 18054039 PMC2746718

[59] Huang GN Thatcher JE McAnally J Kong Y Qi X Tan W. C/EBP transcription factors mediate epicardial activation during heart development and injury. Science. (2012) 338:1599–603. 10.1126/science.1229765 23160954 PMC3613149

[60] Iyer LM Nagarajan S Woelfer M Schoger E Khadjeh S Zafiriou MP. A context-specific cardiac beta-catenin and GATA4 interaction influences TCF7L2 occupancy and remodels chromatin driving disease progression in the adult heart. Nucleic Acids Res. (2018) 46:2850–67. 10.1093/nar/gky049 29394407 PMC5887416

[61] Ko T Fujita K Nomura S Uemura Y Yamada S Tobita T. Quantification of DNA damage in heart tissue as a novel prediction tool for therapeutic prognosis of patients with dilated cardiomyopathy. JACC Basic Transl Sci. (2019) 4:670–80. 10.1016/j.jacbts.2019.05.010 31709317 PMC6834953

[62] Nomura S Satoh M Fujita T Higo T Sumida T Ko T. Cardiomyocyte gene programs encoding morphological and functional signatures in cardiac hypertrophy and failure. Nat Commun. (2018) 9:4435. 10.1038/s41467-018-06639-7 30375404 PMC6207673

[63] Ren Z Yu P Li D Li Z Liao Y Wang Y. Single-cell reconstruction of progression trajectory reveals intervention principles in pathological cardiac hypertrophy. Circulation. (2020) 141:1704–19. 10.1161/CIRCULATIONAHA.119.043053 32098504

[64] Luecken MD Theis FJ. Current best practices in single-cell RNA-seq analysis: a tutorial. Mol Syst Biol. (2019) 15:e8746. 10.15252/msb.20188746 31217225 PMC6582955

[65] Linna-Kuosmanen S Schmauch E Galani K Boix CA Hou L Örd T. Single-cell dissection of live human hearts in ischemic heart disease and heart failure reveals cell-type-specific driver genes and pathways. bioRxiv. (2021). 10.1101/2021.06.23.449672

[66] Nomura S. Single-cell genomics to understand disease pathogenesis. J Hum Genet. (2021) 66:75–84. 10.1038/s10038-020-00844-3 32951011 PMC7728598

[67] Pandelakis M Delgado E Ebrahimkhani MR. CRISPR-based synthetic transcription factors *in vivo*: the future of therapeutic cellular programming. Cell Syst. (2020) 10:1–14. 10.1016/j.cels.2019.10.003 31972154 PMC7175797

[68] Cong L Zhou R Kuo YC Cunniff M Zhang F. Comprehensive interrogation of natural TALE DNA-binding modules and transcriptional repressor domains. Nat Commun. (2012) 3:968. 10.1038/ncomms1962 22828628 PMC3556390

[69] Kundakovic M Chen Y Guidotti A Grayson DR. The reelin and GAD67 promoters are activated by epigenetic drugs that facilitate the disruption of local repressor complexes. Mol Pharmacol. (2009) 75:342–54. 10.1124/mol.108.051763 19029285 PMC2684898

[70] Lanzillotta A Pignataro G Branca C Cuomo O Sarnico I Benarese M. Targeted acetylation of NF-kappaB/RelA and histones by epigenetic drugs reduces post-ischemic brain injury in mice with an extended therapeutic window. Neurobiol Dis. (2013) 49:177–89. 10.1016/j.nbd.2012.08.018 22971966

[71] Brandes RP Dueck A Engelhardt S Kaulich M Kupatt C De Angelis MT. DGK and DZHK position paper on genome editing: basic science applications and future perspective. Basic Res Cardiol. (2021) 116:2. 10.1007/s00395-020-00839-3 33449167 PMC7810637

[72] Xu X Qi LS. A CRISPR-dCas toolbox for genetic engineering and synthetic biology. J Mol Biol. (2019) 431:34–47. 10.1016/j.jmb.2018.06.037 29958882

[73] Hodges CA Conlon RA. Delivering on the promise of gene editing for cystic fibrosis. Genes Dis. (2019) 6:97–108. 10.1016/j.gendis.2018.11.005 31193992 PMC6545485

[74] Min YL Bassel-Duby R Olson EN. CRISPR correction of duchenne muscular dystrophy. Annu Rev Med. (2019) 70:239–55. 10.1146/annurev-med-081117-010451 30379597 PMC6415693

[75] Zhan T Rindtorff N Betge J Ebert MP Boutros M. CRISPR/Cas9 for cancer research and therapy. Semin Cancer Biol. (2019) 55:106–19. 10.1016/j.semcancer.2018.04.001 29673923

[76] Pineda M Moghadam F Ebrahimkhani MR Kiani S. Engineered CRISPR systems for next generation gene therapies. ACS Synth Biol. (2017) 6:1614–26. 10.1021/acssynbio.7b00011 28558198

[77] Alerasool N Segal D Lee H Taipale M. An efficient KRAB domain for CRISPRi applications in human cells. Nat Methods. (2020) 17:1093–6. 10.1038/s41592-020-0966-x 33020655

[78] Nunez JK Chen J Pommier GC Cogan JZ Replogle JM Adriaens C. Genome-wide programmable transcriptional memory by CRISPR-based epigenome editing. Cell. (2021) 184:2503–19 e2517. 10.1016/j.cell.2021.03.025 33838111 PMC8376083

[79] Cheng AW Wang H Yang H Shi L Katz Y Theunissen TW. Multiplexed activation of endogenous genes by CRISPR-on, an RNA-guided transcriptional activator system. Cell Res. (2013) 23:1163–71. 10.1038/cr.2013.122 23979020 PMC3790238

[80] Chavez A Tuttle M Pruitt BW Ewen-Campen B Chari R Ter-Ovanesyan D. Comparison of Cas9 activators in multiple species. Nat Methods. (2016) 13:563–7. 10.1038/nmeth.3871 27214048 PMC4927356

[81] Maeder ML Linder SJ Cascio VM Fu Y Ho QH Joung JK. CRISPR RNA-guided activation of endogenous human genes. Nat Methods. (2013) 10:977–9. 10.1038/nmeth.2598 23892898 PMC3794058

[82] Mali P Aach J Stranges PB Esvelt KM Moosburner M Kosuri S. CAS9 transcriptional activators for target specificity screening and paired nickases for cooperative genome engineering. Nat Biotechnol. (2013) 31:833–8. 10.1038/nbt.2675 23907171 PMC3818127

[83] Perez-Pinera P Kocak DD Vockley CM Adler AF Kabadi AM Polstein LR. RNA-guided gene activation by CRISPR-Cas9-based transcription factors. Nat Methods. (2013) 10:973–6. 10.1038/nmeth.2600 23892895 PMC3911785

[84] Bohm S Splith V Riedmayr LM Rotzer RD Gasparoni G Nordstrom KJV. A gene therapy for inherited blindness using dCas9-VPR-mediated transcriptional activation. Sci Adv. (2020) 6:eaba5614. 10.1126/sciadv.aba5614 32875106 PMC7438099

[85] Liao HK Hatanaka F Araoka T Reddy P Wu MZ Sui Y. *In vivo* target gene activation via CRISPR/Cas9-mediated trans-epigenetic modulation. Cell. (2017) 171:1495–507 e1415. 10.1016/j.cell.2017.10.025 29224783 PMC5732045

[86] Wangensteen KJ Wang YJ Dou Z Wang AW Mosleh-Shirazi E Horlbeck MA. Combinatorial genetics in liver repopulation and carcinogenesis with a *in vivo* CRISPR activation platform. Hepatology. (2018) 68:663–76. 10.1002/hep.29626 29091290 PMC5930141

[87] Zhou H Liu J Zhou C Gao N Rao Z Li H. *In vivo* simultaneous transcriptional activation of multiple genes in the brain using CRISPR-dCas9-activator transgenic mice. Nat Neurosci. (2018) 21:440–6. 10.1038/s41593-017-0060-6 29335603

[88] Matharu N Rattanasopha S Tamura S Maliskova L Wang Y Bernard A. CRISPR-mediated activation of a promoter or enhancer rescues obesity caused by haploinsufficiency. Science. (2019) 363:eaau0629. 10.1126/science.aau0629 30545847 PMC6570489

[89] Peabody DS. The RNA binding site of bacteriophage MS2 coat protein. EMBO J. (1993) 12:595–600. 10.1002/j.1460-2075.1993.tb05691.x 8440248 PMC413242

[90] Xu X Tan X Tampe B Wilhelmi T Hulshoff MS Saito S. High-fidelity CRISPR/Cas9- based gene-specific hydroxymethylation rescues gene expression and attenuates renal fibrosis. Nat Commun. (2018) 9:3509. 10.1038/s41467-018-05766-5 30158531 PMC6115451

[91] Meng X Zheng M Yu M Bai W Zuo L Bu X. Transplantation of CRISPRa system engineered IL10-overexpressing bone marrow-derived mesenchymal stem cells for the treatment of myocardial infarction in diabetic mice. J Biol Eng. (2019) 13:49. 10.1186/s13036-019-0163-6 31164920 PMC6543626

[92] Schoger E Carroll KJ Iyer LM McAnally J Tan W Liu N. CRISPR-mediated activation of endogenous gene expression in the postnatal heart. Circ Res. (2020) 126:6–24. 10.1161/CIRCRESAHA.118.314522 31730408

[93] Chaudhry HW Dashoush NH Tang H Zhang L Wang X Wu EX. Cyclin A2 mediates cardiomyocyte mitosis in the postmitotic myocardium. J Biol Chem. (2004) 279:35858–66. 10.1074/jbc.M404975200 15159393

[94] Pasumarthi KB Nakajima H Nakajima HO Soonpaa MH Field LJ. Targeted expression of cyclin D2 results in cardiomyocyte DNA synthesis and infarct regression in transgenic mice. Circ Res. (2005) 96:110–8. 10.1161/01.RES.0000152326.91223.4F 15576649

[95] Mohamed TMA Ang YS Radzinsky E Zhou P Huang Y Elfenbein A. Regulation of cell cycle to stimulate adult cardiomyocyte proliferation and cardiac regeneration. Cell. (2018) 173:104–16 e112. 10.1016/j.cell.2018.02.014 29502971 PMC5973786

[96] Dominguez AA Lim WA Qi LS. Beyond editing: repurposing CRISPR-Cas9 for precision genome regulation and interrogation. Nat Rev Mol Cell Biol. (2016) 17:5–15. 10.1038/nrm.2015.2 26670017 PMC4922510

[97] Zalatan JG Lee ME Almeida R Gilbert LA Whitehead EH La Russa M. Engineering complex synthetic transcriptional programs with CRISPR RNA scaffolds. Cell. (2015) 160:339–50. 10.1016/j.cell.2014.11.052 25533786 PMC4297522

[98] Gao Y Xiong X Wong S Charles EJ Lim WA Qi LS. Complex transcriptional modulation with orthogonal and inducible dCas9 regulators. Nat Methods. (2016) 13:1043–9. 10.1038/nmeth.4042 27776111 PMC5436902

[99] Inagawa K Miyamoto K Yamakawa H Muraoka N Sadahiro T Umei T. Induction of cardiomyocyte-like cells in infarct hearts by gene transfer of Gata4, Mef2c, and Tbx5. Circ Res. (2012) 111:1147–56. 10.1161/CIRCRESAHA.112.271148 22931955

[100] Qian L Huang Y Spencer CI Foley A Vedantham V Liu L. *In vivo* reprogramming of murine cardiac fibroblasts into induced cardiomyocytes. Nature. (2012) 485:593–8. 10.1038/nature11044 22522929 PMC3369107

[101] Zhou H Morales MG Hashimoto H Dickson ME Song K Ye W. ZNF281 enhances cardiac reprogramming by modulating cardiac and inflammatory gene expression. Genes Dev. (2017) 31:1770–83. 10.1101/gad.305482.117 28982760 PMC5666675

[102] Abad M Hashimoto H Zhou H Morales MG Chen B Bassel-Duby R. Notch inhibition enhances cardiac reprogramming by increasing MEF2C transcriptional activity. Stem Cell Reports. (2017) 8:548–60. 10.1016/j.stemcr.2017.01.025 28262548 PMC5355682

[103] Zhou H Dickson ME Kim MS Bassel-Duby R Olson EN. Akt1/protein kinase B enhances transcriptional reprogramming of fibroblasts to functional cardiomyocytes. Proc Natl Acad Sci USA. (2015) 112:11864–9. 10.1073/pnas.1516237112 26354121 PMC4586885

[104] Liu Z Wang L Welch JD Ma H Zhou Y Vaseghi HR. Single-cell transcriptomics reconstructs fate conversion from fibroblast to cardiomyocyte. Nature. (2017) 551:100–4. 10.1038/nature24454 29072293 PMC5954984

[105] Zhou Y Wang L Vaseghi HR Liu Z Lu R Alimohamadi S. Bmi1 is a key epigenetic barrier to direct cardiac reprogramming. Cell Stem Cell. (2016) 18:382–95. 10.1016/j.stem.2016.02.003 26942853 PMC4779178

[106] Black JB Adler AF Wang HG D'Ippolito AM Hutchinson HA Reddy TE. Targeted epigenetic remodeling of endogenous loci by CRISPR/Cas9-based transcriptional activators directly converts fibroblasts to neuronal cells. Cell Stem Cell. (2016) 19:406–14. 10.1016/j.stem.2016.07.001 27524438 PMC5010447

[107] Chakraborty S Ji H Kabadi AM Gersbach CA Christoforou N Leong KW. A CRISPR/Cas9-based system for reprogramming cell lineage specification. Stem Cell Reports. (2014) 3:940–7. 10.1016/j.stemcr.2014.09.013 25448066 PMC4264059

[108] Dal-Pra S Hodgkinson CP Dzau VJ. Induced cardiomyocyte maturation: cardiac transcription factors are necessary but not sufficient. PLOS ONE. (2019) 14:e0223842. 10.1371/journal.pone.0223842 31622977 PMC6797484

[109] Garry GA Bezprozvannaya S Chen K Zhou H Hashimoto H Morales MG. The histone reader PHF7 cooperates with the SWI/SNF complex at cardiac super enhancers to promote direct reprogramming. Nat Cell Biol. (2021) 23:467–75. 10.1038/s41556-021-00668-z 33941892 PMC8243412

[110] Karakikes I Ameen M Termglinchan V Wu JC. Human induced pluripotent stem cell-derived cardiomyocytes: insights into molecular, cellular, and functional phenotypes. Circ Res. (2015) 117:80–8. 10.1161/CIRCRESAHA.117.305365 26089365 PMC4546707

[111] Gilbert LA Horlbeck MA Adamson B Villalta JE Chen Y Whitehead EH. Genome-scale CRISPR-mediated control of gene repression and activation. Cell. (2014) 159:647–61. 10.1016/j.cell.2014.09.029 25307932 PMC4253859

[112] Sanson KR Hanna RE Hegde M Donovan KF Strand C Sullender ME. Optimized libraries for CRISPR-Cas9 genetic screens with multiple modalities. Nat Commun. (2018) 9:5416. 10.1038/s41467-018-07901-8 30575746 PMC6303322

[113] Yang J Rajan SS Friedrich MJ Lan G Zou X Ponstingl H. Genome-scale CRISPRa screen identifies novel factors for cellular reprogramming. Stem Cell Rep. (2019) 12:757–71. 10.1016/j.stemcr.2019.02.010 30905739 PMC6450436

[114] Rajewsky N Almouzni G Gorski SA Aerts S Amit I Bertero MG. LifeTime and improving European healthcare through cell-based interceptive medicine. Nature. (2020) 587:377–86. 10.1038/s41586-021-03287-8 32894860 PMC7656507

[115] Yan R Fan C Yin Z Wang T Chen X. Potential applications of deep learning in single-cell RNA sequencing analysis for cell therapy and regenerative medicine. Stem Cells. (2021) 39:511–21. 10.1002/stem.3336 33587792

[116] Nishiyama T Bassel-Duby R Olson EN. Toward CRISPR Therapies for cardiomyopathies. Circulation. (2021) 144:1525–7. 10.1161/CIRCULATIONAHA.121.057203 34748394 PMC8580229

[117] Musunuru K Chadwick AC Mizoguchi T Garcia SP DeNizio JE Reiss CW. *In vivo* CRISPR base editing of PCSK9 durably lowers cholesterol in primates. Nature. (2021) 593:429–34. 10.1038/s41586-021-03534-y 34012082

[118] Caplan AL Parent B Shen M Plunkett C. No time to waste—the ethical challenges created by CRISPR. EMBO Rep. (2015) 16:1421–6. 10.15252/embr.201541337 26450575 PMC4641494

[119] Greenfield A. Making sense of heritable human genome editing: scientific and ethical considerations. Prog Mol Biol Transl Sci. (2021) 182:1–28. 10.1016/bs.pmbts.2020.12.008 34175039

[120] Nxumalo Z Takundwa MM Thimiri Govinda Raj DB. Patents, ethics, biosafety and regulation using CRISPR technology. Prog Mol Biol Transl Sci. (2021) 181:345–65. 10.1016/bs.pmbts.2021.01.023 34127200

[121] Lino CA Harper JC Carney JP Timlin JA. Delivering CRISPR: a review of the challenges and approaches. Drug Deliv. (2018) 25:1234–57. 10.1080/10717544.2018.1474964 29801422 PMC6058482

[122] Doench JG Hartenian E Graham DB Tothova Z Hegde M Smith I. Rational design of highly active sgRNAs for CRISPR-Cas9-mediated gene inactivation. Nat Biotechnol. (2014) 32:1262–7. 10.1038/nbt.3026 25184501 PMC4262738

[123] Fu Y Foden JA Khayter C Maeder ML Reyon D Joung JK. High-frequency off-target mutagenesis induced by CRISPR-Cas nucleases in human cells. Nat Biotechnol. (2013) 31:822–6. 10.1038/nbt.2623 23792628 PMC3773023

[124] Haeussler M Schonig K Eckert H Eschstruth A Mianne J Renaud JB. Evaluation of off-target and on-target scoring algorithms and integration into the guide RNA selection tool CRISPOR. Genome Biol. (2016) 17:148. 10.1186/s13059-016-1012-2 27380939 PMC4934014

[125] Hsu PD Scott DA Weinstein JA Ran FA Konermann S Agarwala V. DNA targeting specificity of RNA-guided Cas9 nucleases. Nat Biotechnol. (2013) 31:827–32. 10.1038/nbt.2647 23873081 PMC3969858

[126] Moreno-Mateos MA Vejnar CE Beaudoin JD Fernandez JP Mis EK Khokha MK. CRISPRscan: designing highly efficient sgRNAs for CRISPR-Cas9 targeting *in vivo*. Nat Methods. (2015) 12:982–8. 10.1038/nmeth.3543 26322839 PMC4589495

[127] Pattanayak V Lin S Guilinger JP Ma E Doudna JA Liu DR. High-throughput profiling of off-target DNA cleavage reveals RNA-programmed Cas9 nuclease specificity. Nat Biotechnol. (2013) 31:839–43. 10.1038/nbt.2673 23934178 PMC3782611

[128] Wang T Wei JJ Sabatini DM Lander ES. Genetic screens in human cells using the CRISPR-Cas9 system. Science. (2014) 343:80–4. 10.1126/science.1246981 24336569 PMC3972032

[129] Xu H Xiao T Chen CH Li W Meyer CA Wu Q. Sequence determinants of improved CRISPR sgRNA design. Genome Res. (2015) 25:1147–57. 10.1101/gr.191452.115 26063738 PMC4509999

[130] O'Geen H Henry IM Bhakta MS Meckler JF Segal DJ. A genome-wide analysis of Cas9 binding specificity using ChIP-seq and targeted sequence capture. Nucleic Acids Res. (2015) 43:3389–404. 10.1093/nar/gkv137 25712100 PMC4381059

[131] La Russa MF Qi LS. The new state of the art: Cas9 for gene activation and repression. Mol Cell Biol. (2015) 35:3800–9. 10.1128/MCB.00512-15 26370509 PMC4609748

[132] Daya S Berns KI. Gene therapy using adeno-associated virus vectors. Clin Microbiol Rev. (2008) 21:583–93. 10.1128/CMR.00008-08 18854481 PMC2570152

[133] Uddin F Rudin CM Sen T. CRISPR gene therapy: applications, limitations, and implications for the future. Front Oncol. (2020) 10:1387. 10.3389/fonc.2020.01387 32850447 PMC7427626

[134] Wright AV Sternberg SH Taylor DW Staahl BT Bardales JA Kornfeld JE. Rational design of a split-Cas9 enzyme complex. Proc Natl Acad Sci USA. (2015) 112:2984–9. 10.1073/pnas.1501698112 25713377 PMC4364227

[135] Swiech L Heidenreich M Banerjee A Habib N Li Y Trombetta J. *In vivo* interrogation of gene function in the mammalian brain using CRISPR-Cas9. Nat Biotechnol. (2015) 33:102–6. 10.1038/nbt.3055 25326897 PMC4492112

[136] Chew WL Tabebordbar M Cheng JK Mali P Wu EY Ng AH. A multifunctional AAV-CRISPR-Cas9 and its host response. Nat Methods. (2016) 13:868–74. 10.1038/nmeth.3993 27595405 PMC5374744

[137] Truong DJ Kuhner K Kuhn R Werfel S Engelhardt S Wurst W. Development of an intein-mediated split-Cas9 system for gene therapy. Nucleic Acids Res. (2015) 43:6450–8. 10.1093/nar/gkv601 26082496 PMC4513872

[138] Moretti A Fonteyne L Giesert F Hoppmann P Meier AB Bozoglu T. Somatic gene editing ameliorates skeletal and cardiac muscle failure in pig and human models of Duchenne muscular dystrophy. Nat Med. (2020) 26:207–14. 10.1038/s41591-019-0738-2 31988462 PMC7212064

[139] Hu Z Wang S Zhang C Gao N Li M Wang D. A compact Cas9 ortholog from Staphylococcus Auricularis (SauriCas9) expands the DNA targeting scope. PLoS Biol. (2020) 18:e3000686. 10.1371/journal.pbio.3000686 32226015 PMC7145270

[140] van Haasteren J Li J Scheideler OJ Murthy N Schaffer DV. The delivery challenge: fulfilling the promise of therapeutic genome editing. Nat Biotechnol. (2020) 38:845–55. 10.1038/s41587-020-0565-5 32601435

[141] Hansen-Bruhn M de Avila BE Beltran-Gastelum M Zhao J Ramirez-Herrera DE Angsantikul P. Active intracellular delivery of a Cas9/sgRNA complex using ultrasound-propelled nanomotors. Angew Chem Int Ed Engl. (2018) 57:2657–61. 10.1002/anie.201713082 29325201

[142] Ju E Li T Ramos da Silva S Gao SJ. Gold nanocluster-mediated efficient delivery of Cas9 protein through pH-induced assembly-disassembly for inactivation of virus oncogenes. ACS Appl Mater Interfaces. (2019) 11:34717–24. 10.1021/acsami.9b12335 31469541 PMC6763369

[143] Yeh WH Chiang H Rees HA Edge ASB Liu DR. *In vivo* base editing of post-mitotic sensory cells. Nat Commun. (2018) 9:2184. 10.1038/s41467-018-04580-3 29872041 PMC5988727

[144] Zhou W Cui H Ying L Yu XF. Enhanced cytosolic delivery and release of CRISPR/Cas9 by black phosphorus nanosheets for genome editing. Angew Chem Int Ed Engl. (2018) 57:10268–72. 10.1002/anie.201806941 29939484

[145] Mullard A. Gene-editing pipeline takes off. Nat Rev Drug Discov. (2020) 19:367–72. 10.1038/d41573-020-00096-y 32415249

[146] Zhang MM Bahal R Rasmussen TP Manautou JE Zhong X-B. The growth of siRNA-based therapeutics: updated clinical studies. Biochem Pharmacol. (2021) 189:114432. 10.1016/j.bcp.2021.114432 33513339 PMC8187268

[147] He Y Khan S Huo Z Lv D Zhang X Liu X. Proteolysis targeting chimeras (PROTACs) are emerging therapeutics for hematologic malignancies. J Hematol Oncol. (2020) 13:103. 10.1186/s13045-020-00924-z 32718354 PMC7384229

[148] Zorn JA Wells JA. Turning enzymes ON with small molecules. Nat Chem Biol. (2010) 6:179–88. 10.1038/nchembio.318 20154666

[149] Henley MJ Koehler AN. Advances in targeting ‘undruggable’ transcription factors with small molecules. Nat Rev Drug Discov. (2021) 20:669–88. 10.1038/s41573-021-00199-0 34006959

[150] Khajanchi N Saha K. Controlling CRISPR with small molecule regulation for somatic cell genome editing. Mol Ther. (2021). 10.1016/j.ymthe.2021.06.014 34174442 PMC8753294

[151] Zhuo C Zhang J Lee JH Jiao J Cheng D Liu L. Spatiotemporal control of CRISPR/Cas9 gene editing. Signal Transduct Target Ther. (2021) 6:238. 10.1038/s41392-021-00645-w 34148061 PMC8214627

[152] Nihongaki Y Yamamoto S Kawano F Suzuki H Sato M. CRISPR-Cas9-based photoactivatable transcription system. Chem Biol. (2015) 22:169–74. 10.1016/j.chembiol.2014.12.011 25619936

[153] Polstein LR Gersbach CA. A light-inducible CRISPR-Cas9 system for control of endogenous gene activation. Nat Chem Biol. (2015) 11:198–200. 10.1038/nchembio.1753 25664691 PMC4412021

[154] Maji B Gangopadhyay SA Lee M Shi M Wu P Heler R. A high-throughput platform to identify small-molecule inhibitors of CRISPR-Cas9. Cell. (2019) 177:1067–79 e1019. 10.1016/j.cell.2019.04.009 31051099 PMC7182439

[155] Balboa D Weltner J Eurola S Trokovic R Wartiovaara K Otonkoski T. Conditionally stabilized dCas9 activator for controlling gene expression in human cell reprogramming and differentiation. Stem Cell Rep. (2015) 5:448–59. 10.1016/j.stemcr.2015.08.001 26352799 PMC4618656

[156] Hazelbaker DZ Beccard A Angelini G Mazzucato P Messana A Lam D. A multiplexed gRNA piggyBac transposon system facilitates efficient induction of CRISPRi and CRISPRa in human pluripotent stem cells. Sci Rep. (2020) 10:635. 10.1038/s41598-020-57500-1 31959800 PMC6971260

[157] Weltner J Balboa D Katayama S Bespalov M Krjutskov K Jouhilahti EM. Human pluripotent reprogramming with CRISPR activators. Nat Commun. (2018) 9:2643. 10.1038/s41467-018-05067-x 29980666 PMC6035213

[158] Wu Y Yang L Chang T Kandeel F Yee JK. A small molecule-controlled Cas9 repressible system. Mol Ther Nucleic Acids. (2020) 19:922–32. 10.1016/j.omtn.2019.12.026 32000033 PMC7063486

[159] Li A Tanner MR Lee CM Hurley AE De Giorgi M Jarrett KE. AAV-CRISPR gene editing is negated by pre-existing immunity to Cas9. Mol Ther. (2020) 28:1432–41. 10.1016/j.ymthe.2020.04.017 32348718 PMC7264438

[160] Gangopadhyay SA Cox KJ Manna D Lim D Maji B Zhou Q. Precision control of CRISPR-Cas9 using small molecules and light. Biochemistry. (2019) 58:234–44. 10.1021/acs.biochem.8b01202 30640437 PMC6586488

